# Influence of Missing Values Substitutes on Multivariate Analysis of Metabolomics Data

**DOI:** 10.3390/metabo4020433

**Published:** 2014-06-16

**Authors:** Piotr S. Gromski, Yun Xu, Helen L. Kotze, Elon Correa, David I. Ellis, Emily Grace Armitage, Michael L. Turner, Royston Goodacre

**Affiliations:** 1 School of Chemistry, Manchester Institute of Biotechnology, The University of Manchester, 131 Princess Street, Manchester M1 7DN, UK; E-Mails: piotr.gromski@postgrad.manchester.ac.uk (P.S.G.); Yun.Xu-2@manchester.ac.uk (Y.X.); helen.kotze@postgrad.manchester.ac.uk (H.L.K.); E.S.Correa@manchester.ac.uk (E.C.); D.Ellis@manchester.ac.uk (D.I.E.); emily.armitage@ceu.es (E.G.A.); 2 School of Chemistry, Brunswick Street, The University of Manchester, Manchester M13 9PL, UK. E-Mail: Michael.Turner@manchester.ac.uk (M.L.T.)

**Keywords:** missing values, metabolomics, unsupervised learning, supervised learning

## Abstract

Missing values are known to be problematic for the analysis of gas chromatography-mass spectrometry (GC-MS) metabolomics data. Typically these values cover about 10%–20% of all data and can originate from various backgrounds, including analytical, computational, as well as biological. Currently, the most well known substitute for missing values is a mean imputation. In fact, some researchers consider this aspect of data analysis in their metabolomics pipeline as so routine that they do not even mention using this replacement approach. However, this may have a significant influence on the data analysis output(s) and might be highly sensitive to the distribution of samples between different classes. Therefore, in this study we have analysed different substitutes of missing values namely: zero, mean, median, k-nearest neighbours (kNN) and random forest (RF) imputation, in terms of their influence on unsupervised and supervised learning and, thus, their impact on the final output(s) in terms of biological interpretation. These comparisons have been demonstrated both visually and computationally (classification rate) to support our findings. The results show that the selection of the replacement methods to impute missing values may have a considerable effect on the classification accuracy, if performed incorrectly this may negatively influence the biomarkers selected for an early disease diagnosis or identification of cancer related metabolites. In the case of GC-MS metabolomics data studied here our findings recommend that RF should be favored as an imputation of missing value over the other tested methods. This approach displayed excellent results in terms of classification rate for both supervised methods namely: principal components-linear discriminant analysis (PC-LDA) (98.02%) and partial least squares-discriminant analysis (PLS-DA) (97.96%) outperforming other imputation methods.

## 1. Introduction

Metabolomics is the study of all metabolites in a biological system under a given set of conditions [1]. The classical technologies for the analysis of metabolites (*i.e.*, chemical entities) include: Gas chromatography-mass spectrometry (GC-MS), liquid chromatography-mass spectrometry (LC-MS), capillary electrophoresis-mass spectrometry (CE-MS), nuclear magnetic resonance (NMR) spectroscopy, Fourier transform-infrared (FT-IR) spectroscopy, and many more [2]. These methods have been widely applied in countless metabolomics research studies. These include for example: cell line studies probing intracellular metabolites [3]; animal models for cancer study, such as mouse tumours [4]; as well as general metabolic profiling for the analysis of human serum [5]. Unfortunately not all of these methods generate a complete data set, for instance GC-MS and LC-MS analyses employ chromatographic separation prior to MS and thus require a complex deconvolution step to transform these 3D data matrices into lists of annotated features (metabolites) with (relative) abundances. The result of this process is often a matrix that is not fully populated and thus presents a major problem in data processing due to these missing values [6].

We believe that one of the most important steps in GC-MS data pre-processing of metabolomics data, next to peak detection and alignment, is handling missing values [7]. In our experience, typically 10%–20% of GC-MS data from an experiment are missing, with the same level of missing values also reported in many other studies (e.g., Hrydziuszko and Viant (2011) [8] and Xia *et al.* (2009) [9]). Whilst it is obvious that a missing value is when a matrix contains an empty cells usually recorded as “Not a Number” (NaN) or more worryingly as a zero which cannot be readily distinguished from the real absence of a feature rather than a failure in the analysis. The obvious question that arises from these observations is: What are the roots of these missing values? Prior to any analysis it is good practice to identify the origins of missing values whether they are truly missing or not [10]. Missing values may arise due to various reasons, such as: (1) limits in computational detection; (2) imperfection of the algorithms whereby they fail in the identification of some of the signals from the background; (3) low intensity of the signals used; (4) measurement error; and finally (5) deconvolution that may result in false negative during separation of overlapping signals [6,8,10,11,12,13,14,15,16,17].

Currently, the most well-known substitute for missing values is their substitution with a mean value [6]. In fact, some researchers do not specifically state how this aspect of data analysis in their metabolomics pipeline has been performed and use this replacement approach as a common practice. This is despite the fact that this problem has been well recognised in the literature as an important aspect and also appears within the minimum reporting standards for data analysis for metabolomics [7]. Methods which have been reported in the literature include: replacing missing values by half of the minimum value found in the data set [9]; missing value imputation using probabilistic principal component analysis (PPCA) [15], Bayesian PCA (BPCA) [18] or singular value decomposition imputation (SVDImpute) [9]; replacing missing value by means of nearest neighbours [6]; or replacing the missing values with zeros [19].

Whilst many unsupervised and supervised learning for the analysis of high dimensional metabolomics data require a complete dataset [2,6,7,20]. Consequently, there is a need to analyse and identify correct approaches for the replacement of missing values. Nevertheless, this seemingly important aspect of data pre-processing has not received wide attention in this field. Herein, we investigate this problem using a common set of metabolomics data produced using GC-MS which contained ~15% missing values, where the goal of the study was to analyse cancer cell lines in terms of changes in oxygen level and which metabolite features change during this process [21]. Therefore, we evaluated five relatively simple potential missing values substitutes—zero, mean, median, k-nearest neighbours (kNN) [22] and random forest (RF) [23] replacements—in terms of their influence on unsupervised and supervised learning and thus their impact on the final output(s); these outputs are related to cluster compactness from replicate biological measurements. Moreover, to our knowledge these approaches have not been compared directly for the analysis of GC-MS data.

## 2. Experimental Section

### 2.1. Materials and Methods

#### 2.1.1. Cell Culture and Experimental Protocol

The methods used for cell culture have previously been described [21]. Experimental analysis proceeded as follows: MDA-MB-231 cells were seeded and allowed to adhere for 24 h in 95% air and 5% CO_2_. Cells were divided into three groups: one group was placed in a 95% air and 5% CO_2_ incubator (normoxia); one group placed in a 1% O_2_, 5% CO_2_ balanced with N_2_ hypoxybox (hypoxia) and one group was placed in an anoxic chamber (Bactron anaerobic chamber, Sheldon Manufacturing, Cornelius, OR, USA) where 5% CO_2_, 5% H_2_ and 90% N_2_ (BOC, Manchester, UK) was flowed over a palladium catalyst to remove any remaining oxygen (anoxia) for 24 h. Each of the three groups were split into three sub groups, which were dosed with 0, 0.1 or 1 µM doxorubicin for 16 h whilst remaining in the predefined oxygen condition for a further 24 h.

#### 2.1.2. Methanol Metabolite Extraction

Metabolites were extracted whilst cells remained in the predefined oxygen condition. The hypoxybox was sealed and cycled into the anoxic chamber prior to the extraction of metabolites. Extracellular medium was decanted and cells were washed with three 1 mL aliquots of PBS. A total of 1 mL of methanol (−48 °C) was added to each dish to quench the metabolism [24]. Cell scrappers detached adherent cells from the Petri dish allowing the intracellular metabolites to be extracted into the methanol. The solution was transferred into a pre-weighed Eppendorf tube followed by three freeze thaw cycles (frozen using liquid nitrogen). Cells were centrifuged at 17,000 × *g* for 15 min to separate the pellet from the supernatant. The supernatant was transferred into a fresh Eppendorf tube and the pellet was lyophilised and weighed. The volume of supernatant lyophilised was normalised according to the dry weight of the pellet for intracellular samples. A QC was produced using 150 µL of remaining supernatant from each sample. Aliquots (1 mL) of the QC solution were placed into Eppendorf tubes. Both the sample and QC were lyophilised.

#### 2.1.3. Metabolic Profiling Using GC-MS and Raw Data Processing

A total of 8 randomised batches were analysed using GC-MS. Of these batches, 7 contained 40 samples and 9 QCs each and 1 batch contained 30 samples and 9 QCs. Samples were analysed on an Agilent 6890 GC (Agilent Technologies, Stockport, UK) coupled to a LECO Pegasus III (Leco Corp., St. Joseph, MO, USA) EI-ToF-MS. The GC-MS instrument setup used has been previously described [5,25]. QCs were used to condition the GC-MS before sample injection. A total of 5 injections of QC samples were performed prior to analysing samples to normalise the chromatographic conditions to the sample matrix. Every 5 sample injections were followed with a single QC injection. In addition, three QC injections were made at the end of each analysis. These QC samples were compared to account for the system stability in each batch analysed. Briefly, the temperature was set at 70 °C for 4 min followed by a 20 °C increase every min until 300 °C was reached and stabilised for 4 min. Samples (2 μL) were injected onto the column. The mass spectrometer was operated from *m/z* 45-600. The total duration per sample was 25 min.

The processing of raw GC-MS data were performed following the methods described previously, using the LECO ChromaToF v3.25 software package to apply the chromatographic deconvolution algorithm [5,25]. Chromatographic peaks from the QC samples selected from each analytical block were manually integrated and added to a database, which included the peak area, the retention index and mass spectrum to compile a reference database. This reference database was manually inspected to ensure the mass spectra had the required fragmentation patterns and met the criteria for signal-to-noise (*S/N*) ratio and chromatographic peak width. Metabolites with fewer than 50% of features detected in the QC were removed [26].

Identification of metabolites within the compiled database was performed through searching against an in-house mass spectral and retention index library as described previously, where a high confidence match was assigned when the mass spectral match was greater than 80% and the retention index was within 30 [27]. All other matches that did not meet this specification were not regarded as a high confidence match. The metabolite library was previously developed in-house from authentic standards at the University of Manchester. Metabolites that were not identified in the in-house database were searched against the Golm metabolome database [28] and a similar match score to the in-house database was applied. 

In this dataset, 102 peaks were integrated from the GC chromatogram of which 45% were unidentified peaks; these unidentified peaks were removed from the data matrix. The coefficient of variation (CV) was calculated for metabolites detected in QC samples. Two metabolites with CV > 30% were removed from the whole data matrix as these were considered to have poor reproducibility [26]. We followed the metabolite identification protocols outlined by the metabolomics standards initiative [29] where the in-house matches are MSI level 1 (43 metabolites) and all other identification in this manuscript to MSI level 2 (9 metabolites).

Finally the GC-MS data were normalised to the internal standard succinic *d*_4_ acid to account for the technical variability associated with chemical derivatisation and low sample injection volumes. The chromatogram peaks areas were exported as an ASCII file into Microsoft Excel^®^ and these 52 metabolites were used for all further analyses.

### 2.2. Software Tools

All data analyses used in this work were conducted in R version 2.15.0 and 3.1.0; R is a software environment [30] that contains a variety of packages useful for data analysis and is a free open source program [30]. The following R packages were employed for visualization and statistical analysis; “chemometrics” [31] package has been used to determine the number of principal components (PCs) with cross-validation and for hierarchical cluster analysis (HCA) and picturing; “MASS” package was used for linear discriminant analysis (LDA) [32]; “rgl” package was applied as a 3D visualization device [33]; “mixOmics” [34] package was used to perform both PCA and partial least squares-discriminant analysis (PLS-DA); package “impute” [35] which is a part of Bioconductor an open source and open development software project to provide tools for the analysis and comprehension of genomic data [36] was applied for substitution of missing values using kNN; and finally “missForest” [23,37] for missing value imputation using RF.

### 2.3. Data Preparation

Data pre-treatment is a very important process because of its ability to make the data clearer and suited to analysis [38]. Here, we used autoscaling as it is the probably most reliable method [7,39]. This was conducted after replacing missing values. In autoscaling for each column (input feature) the mean value of that column is subtracted, followed by dividing the row entries for that column by the standard deviation within the same column. In other words, the aim of autoscaling is to break down relation with irregularities in order to produce slighter, well-structured relations, which in practice allow for better data recognition [39,40].

### 2.4. Imputation Methods

In this study five different imputation methods of missing value were applied: zero, mean, median, kNN and RF. Zero imputation was used to replace all missing value with zeros. Mean imputation to replace the missing values with average value of corresponding metabolites (variables). The median imputation corresponds to central value of metabolite that split its values into two equal parts the higher and the lower part. 

#### 2.4.1. Imputation of Missing Values Using k-Nearest Neighbours (kNN).

kNN for each metabolite identifies missing values by recognizing the nearest neighbours of that feature using Euclidean distance [22]. The calculation takes into consideration that each candidate neighbours might miss some of the coordinates. As a result, the method uses the average distance from the non-missing locations. As long as the k nearest neighbours for each metabolite is identified, the imputation of missing value is computed by averaging of non-missing values of its neighbours. Caution need to be taken when one want to deal with the cases where all of the neighbours are missing in a particular group. Nevertheless, this obstacle is circumvented by taking the overall column mean for that particular metabolite. Consequently, the technique has the ability to deal with the data set that contains large number of variables that include missing values [22].

#### 2.4.2. Missing Value Imputation Using Random Forest (RF)

RF is a classification and regression technique that can handle both parametric and non-parametric data sets of complex linear and non-linear problems [41]. The approach is based on the estimation of imputation error which is calculated for the bootstrapped samples and therefore no separate cross-validation is required. The process begins with splitting the missing data into training and test sets. The training set is used to generate a group of trees for the observed values in the training data set to predict the missing values. Consequently, the first step is to replace each of the missing data points from training set with a mean of the observed values for that variable. All missing values are replaced and the proximity matrix is calculated for first iteration set. The average weights calculated from the proximity matrix for first iteration are used to replace the missing values in the second iteration and so on until the stopping criterion is satisfied. The stopping criterion is passed based on the difference between the first and second iteration, then between second and third and so on. This is achieved when both differences have become larger once. As soon as this is accomplished the final matrix with replaced values is used to perform further analysis [23].

### 2.5. Unsupervised Learning

Unsupervised learning algorithms attempt to find hidden structure in data, which displays any similarities between data points (e.g., metabolite levels), such as how close they are to each other and whether the data contain any outliers [42]. Additionally, these so-called exploratory methods are mainly based on cluster analysis. Cluster analysis aims to cluster data (*i.e.*, samples) into constituent groups which describe common characteristics that they possess. These clusters should display high internal homogeneity within clusters (or as high as possible), and high external heterogeneity between clusters.

#### 2.5.1. Principal Components Analysis (PCA)

The most frequently used exploratory analysis method is principal component analysis (PCA) which is used for computing linear latent variables (components). PCA is possibly one of the oldest and most well-known methods of multivariate data analysis (MVA) method which was invented in 1901 by Karl Pearson [43]. This technique is one of the simplest multivariate statistical procedures that reduce the dimensionality of data sets comprised of a large number of possibly correlated variables. On finding these correlated observations, Hotelling showed that PCA retains these features allowing any variability present in the data to be explained and visualized [44]. In general, the correlated original variables are transformed into a set of new uncorrelated variables, (principal components (PCs)), ordered according to their decreasing variance, whereby the first PCs describes more variability than any other PC. The absence of correlation is a valuable property because it means that these indices (PCs) are assessing dissimilar correlations in the data [45].

Rather than generate a single PCA model we used *k*-fold cross-validation in order [46,47]: (i) to generate a generalized scree plot which allows us to assess random sampling of the population; and (ii) to calculate how many times the 3 groups were recovered (for this we calculate Q^2^ based on the test sets from the *k*-folds). Thus for PCA we applied 10-fold cross-validation and we repeated this 100 times. This allowed the explained variance for each model to be assessed. These was performed to determine the optimal number of PCs and from 1 to *n* PCs were used in this process and the overall percent explain variance represented as a box-whisker plot; further explanation of the PCA procedure can be found in supplementary information (SI).

#### 2.5.2. Hierarchical Cluster Analysis (HCA)

There are numerous clustering approaches such as partitioning clustering which is stepwise process that work by partitioning observations, optimization-partitioning which allows for reassignment of objects to optimize an overall parameter, density or mode-seeking methods where cluster are created based on dense concentration of entities, and clumping with possible overlapping samples. However, in this study we have selected the most commonly used method, namely hierarchical clustering analysis (HCA) [48,49,50]. This approach is a fast and reliable technique, allowing for a huge amount of closely related samples or features to be organized into separated clusters [50].

Joining of clusters is based on similarities and dissimilarities in metabolites that allow for the separation of groups, with the most important factor in the creation of cluster beings the distance between two objects. Distances in clustering can be measured in various ways. One of the most common and frequently used is Euclidean distance. However, this can be only used to measure the distance between samples, but not a group of samples constructing clusters. Therefore, here in this study “Ward” linkage was used to link groups of different clusters together and provides a clear visualization of the results [49,50,51,52]; further explanation of the HCA can be found in SI.

In general, unsupervised learning is applied when there are no unlabeled training data (*i.e.*, the modeling is performed on only the X data (SI)). On the other hand, there are supervised techniques that are mainly focused on classification of an input signal, which is to be defined as a member of predefined classes (so called Y data (SI)).

### 2.6. Supervised Learning

Supervised learning might be represented by a variety of methods and a particularly popular method used is discriminant analysis. By way of an example we can consider the scenario where we want to use supervised methods to classify whether cell samples under investigation by GC-MS metabolomics are cancerous or not. In this process a model is first constructed (calibrated) using a data training set of X data (metabolite levels) and Y data (cancer *versus* control) whose identities are unequivocally known. Obviously, it is essential to establish the training set prior to any analysis and this must be fully representative of the problem domain [53].

#### 2.6.1. Linear Discriminant Analysis (LDA)

Linear discriminant analysis (LDA) (also known as discriminant function analysis (DFA)) [54] is a statistical tool for studying the association between a set of predictors *versus* a categorical response. This method aims to find the best linear separation between groups by determining the minimum dimensions at which groups can be separated. LDA is used in statistics as a dimensionality reduction technique in many classification problems, as well as being a useful variable selection method. In general, LDA is a procedure to find a linear combination of features, which describe or separate two or more groups of samples. In other words, LDA classifies data by providing more distinguishable classes using multivariate decision boundaries [54]. LDA can be used without prior PCA if the following condition is satisfied [38,55,56,57]:

(*N_s_* − *N_𝑔_* − 1) > *N_v_* (1)

where *N_s_* correspond to number of samples, *N_g_* to the number of groups, and *N_v_* reflects the number of inputs (features).

However, LDA cannot be used with data which are highly collinear, such as is the case in the present study. Therefore, in this investigation PCA was performed prior to LDA to remove the effect of collinearity and reduce the number of variables in the X data matrix.

#### 2.6.2. Partial Least Squares-Discriminant Analysis (PLS-DA)

Another widely used statistical tool for classification is partial least squares-discriminant analysis (PLS-DA) [58] which can be used either for both classification and variable selection [59]. The approach is an extension of the traditional PLS regression model which was developed for quantitative predictions. The main difference is that in PLS-DA, a “dummy” response matrix was used to code multiple classes and the algorithm is calibrated to predict group membership. The maximum separation between classes (groups) is established by simultaneous rotation of the X and Y components [60,61].

### 2.7. Model Validation

To assess the classification rates for PC-LDA and PLS-DA for each of the five missing value imputations we performed bootstrapping (with replacement). This approach proposed by Efron was used to minimize the risk of misrepresentative results as a single training set—test set split may not be sufficient enough to draw valid conclusions. Bootstrapping was performed 100 times and on average training sets will contain ~63.2% and test sets ~36.8% of all samples [62,63]. Therefore, the number of components for both PLS-DA and PC-LDA has been estimated based on percentage of correct classification in validation.

## 3. Results and Discussion

As discussed above in order to compare five different missing value substitutes we used data that had been generated from the metabolic profiling of MDA-MB-231 breast cancer cells cultured in three oxygen levels—normoxia, hypoxia and anoxia—that had been treated with 0.1 or 1 μM doxorubicin drug, along with a control where no drug was used. Previously, for this data set the following analyses were performed: two-way ANOVA, PCA, PC-DFA, and correlation analysis [21,64].

In this study for the first approach we used the full data set which included all classes. These were 3 aeration environments times 3 drug doses: (1) normoxia; (2) normoxia + 0.1 μM doxorubicin; (3) normoxia + 1 μM doxorubicin; (4) hypoxia; (5) hypoxia + 0.1 μM doxorubicin; (6) hypoxia + 1 μM doxorubicin; (7) anoxia; (8) anoxia + 0.1 μM doxorubicin; and finally (9) anoxia + 1 μM doxorubicin. Initially we looked at PCA and the discriminant analyses on these 9 groups. However, due to the relatively large number of groups this separation is very difficult to visualize as is illustrated in SI Figure S3. To try and uncouple the environment and the drug dosing we applied multiblock PCA (see SI for details) that should provide us with a graphical overview of separation between these two different projections [65]. However, this approach did not work very well (see SI Figure S4). Therefore, our analysis was performed for three different environments, as the effects of normoxia, hypoxia or anoxia were the dominating phenotype.

Visualization of the scores generated from unsupervised and supervised methods is the main tool used in this study for evaluating the effect of the missing value substitutes (zero, mean, median, kNN and RF). The general idea being that for the three different groups these should group close to each other and away from the other groups. That is to say, (i) more compact clusters are more desirable; and (ii) the greater the distance between clusters is also a preferred outcome.

**Figure 1:**
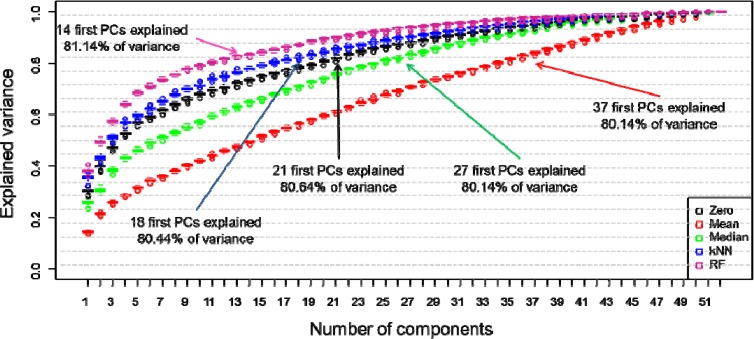
Scree plot showing the effect of the five different missing value substitutes—zero (black boxplots), mean (red boxplots) and median (green boxplots), k-nearest neighbours (kNN) (blue boxplots) and RF (violet boxplots)—on the total explained variance recovered in principal component analysis (PCA). 10-fold cross-validation was repeated 100 times and the boxplots display statistical distribution among all 100 iterations. The arrows indicate the minimum number of components that are required to explain >80% variance.

For the first analysis we performed PCA and evaluated the total explained variance (TEV) captured in the principal components (PCs). Rather than doing this on all of the data in a single experiment we used 10-fold cross-validation, where 9 folds were used to generate each PCA and the 10th fold was left out, this was repeated until each fold had been left out once; this process was repeated 100 times with random splits into the 10 different partitions/folds. Figure 1 is a scree plot which illustrates how each of the different substitution can influence variance across the analysis. Here in this study 80% cut off point has been selected as criterion in estimation of the number of PCs to be used. However, this might vary from study to study and depends on the amount of information that needs to be included in the model. As can be observed in Figure 1 when RF was applied as a substitution of missing value only 1–14 PCs were required to cover over 80% of explained variance. This result is followed by kNN method where 1–18 PCs where needed to cover similar level of explained variance. For zero replacement 1–21 PCs were used to reach comparable level of variance this is followed by median with 1–27 PCs were required to cover 80% of explained variance, and the mean replacement approach required the highest number and the first 37 PCs (out of a possible 52) were needed to cover same level of variance. As ~15% of the data were zeros it is not surprising that such good results for zero were found as the variance will obviously be lower. What is interesting is that the median captures more variance than the mean imputation approach. Indeed this is very evident in just PC1 where for zero and median the first PCs characterize ~30% TEV whilst the mean explains <20%. Such a good results for RF might originate from the fact that the methods do not take into consideration the samples distributions.

**Figure 2:**
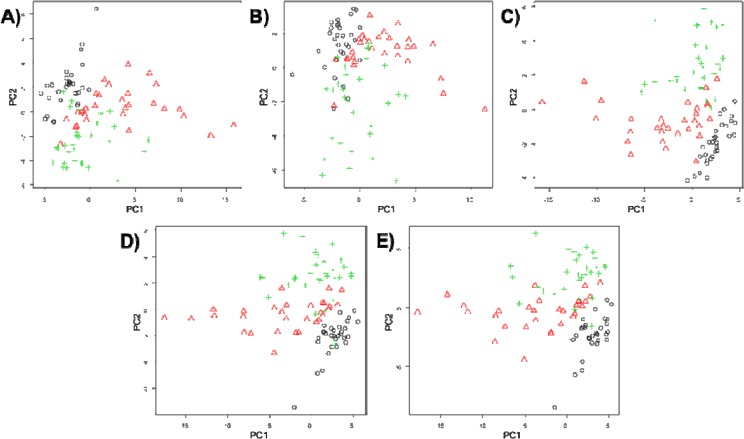
PCA scores plots showing comparisons of five different missing value substitutes on for: (**A**) zero; (**B**) mean; (**C**) median; (**D**) kNN; and (**E**) RF. Symbols represent: normoxia (black circles), hypoxia (red triangles) and anoxia (green pluses).

Having established the shape of the PCA plots for the five different imputation tenuis of the Coral Sea. methods, the scores plots for PC1 *vs.* PC2 were generated when all the metabolomics data was used (Figure 2). 3D plots of PC1 *vs.* PC2 *vs.* PC3 are available in SI (Figure S5). Visual inspection of these PC scores plots indicates that despite some overall in all five plots, the separations is not quite as good for zero and mean imputation, whereas the median, kNN and RF replacement results in reasonably good separation between three groups.

**Figure 3:**
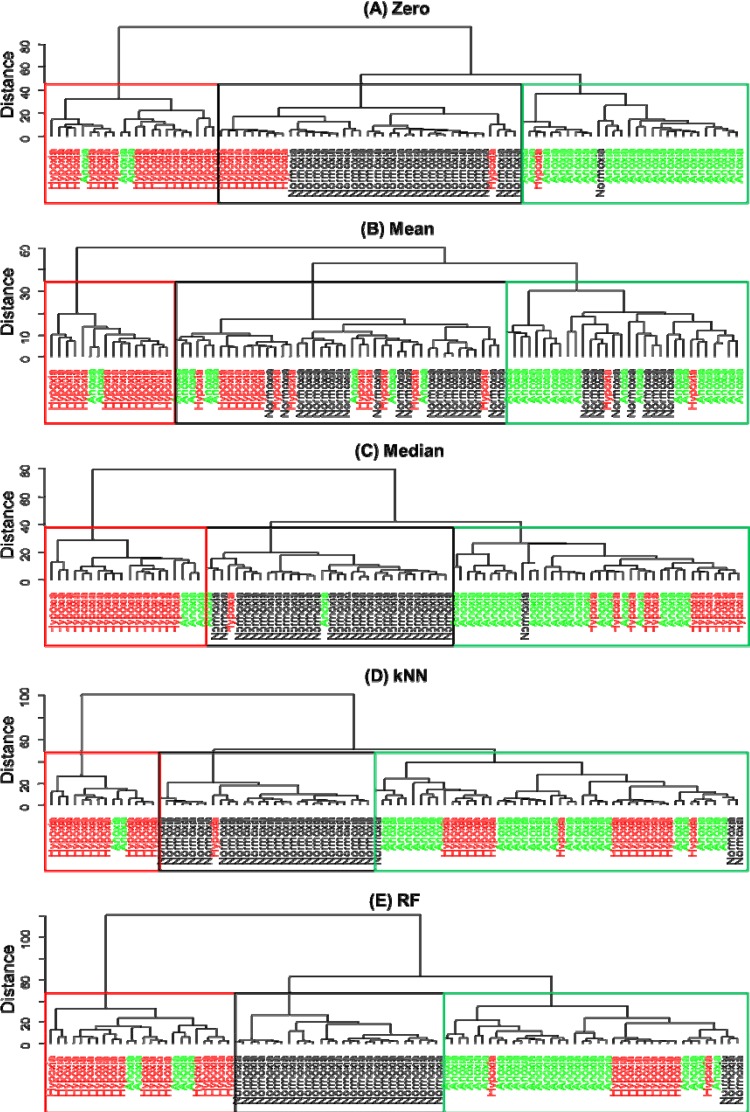
HCA based on Wards linkage for five different value substitutes: (**A**) zero; (**B**) mean; (**C**) median; (**D**) kNN; and (**E**) random forest (RF). Boxes indicate where the majority of the following sample types are located. Text are coloured according to normoxia (black), hypoxia (red) or anoxia (green).

Figure 3 shows dendrograms based on Euclidean distance and “Wards” linkage. Note that these dendrogram can be used to characterise particular clustering and the distance gives an element of multilevel hierarchy [49]. To identify grouping within the dendrogram we have coloured the text according to the atmospheric environment in which the cells were cultivated and used boxes to show where the majority of the same samples fall.

From the HCA we can see that different substitutes of missing values have a significant impact on clustering and it is clear than when the mean is used to replace the missing values that the clustering does not reflect the three expected groups. By contrast, all other replacement methods generate dendrograms that more-or-less recover the three environmental condition groups, with obvious exceptions that can be seen in Figure 3. This can also be observed in Table S1 (SI) that represent a purity measure of HCA; the percentage of samples coming from the same class as one cluster.

Although PCA and HCA are useful because they are unsupervised analyses methods the interpretation is somewhat subjective as cluster plots and dendrograms are used. Moreover, the interpretation is after the cluster code is broken (*i.e.*, the labels or colours are put onto the clusters) and this is not really appropriate for providing information about classification rate. Therefore, in the next step we investigated the impact of the different missing value replacements on supervised methods based on discriminant analyses.

**Figure 4:**
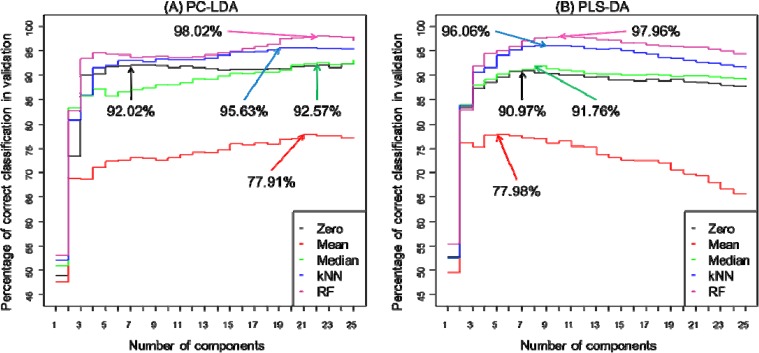
Comparison of prediction accuracy for normoxia, hypoxia and anoxia when five different missing value substitutes (zero, mean, median, kNN and RF) are used in (**A**) principal component-linear discriminant analysis or (**B**) partial least squares-discriminant analysis. The results are displayed as an average percentage of correct classification in the test sets from 100 bootstraps *versus* the number of PCs used for. The arrows indicate first local maxima at which an optimum classification rate has been accomplished. Lines are coloured according to zero (black), mean (red), median (green), kNN (blue) or RF (violet) imputations.

As described above bootstrapping was used to construct 100 different PC-LDA or PLS-DA models and inspection of these allows one to predict how accurate classification is. For PC-LDA this is in terms of recovery of test data with appropriate training data in LDA scores space, whilst for PLS-DA the Y predicted matrix is inspected and the binary encoding in this PLS2 model used. That is to say the encoding for normoxia is 100, hypoxia is 010 and for anoxia the output would be 001. Figure 4 highlights how the different replacement approaches can have an impact on the average correct classification rates of the 100 models for PC-LDA (Figure 4A) and for PLS-DA (Figure 4B) and also shows the effect of the number of components used on this classification accuracy. To aid interpretation this plot is annotated with the points at which the highest classification occurs. For PC-LDA the preferred model is the one with RF imputation demonstrated an excellent classification rate of 98.02% with 22 latent variables. In addition, this method performed the best along all components in comparison to other replacement techniques. Second best result of 95.63% classification rate was recorded for kNN. Zero imputation as whilst this is slightly lower (92.02%) than the median imputation (92.57%), however fewer latent variables are used, 7 compared with 22 PCs. Whilst for PLS-DA both zero and median imputation give very similar predictive abilities (90.97% *vs.* 91.76%) and use a very similar number of latent variables (7 *vs.* 8). In terms of best performance for PLS-DA yet again RF (97.96%) and kNN (96.06%) accomplished the best in comparison to other methods; however the difference between both is smaller as for PC-LDA. What is clear from both PC-LDA and PLS-DA is that mean imputation (Figure 4) is significantly worse and predictions are only ~78%. However, these differences in classification rates are very small as it could be expected as these both methods should display similar results as reported in the literature [7,58,66].

**Figure 5:**
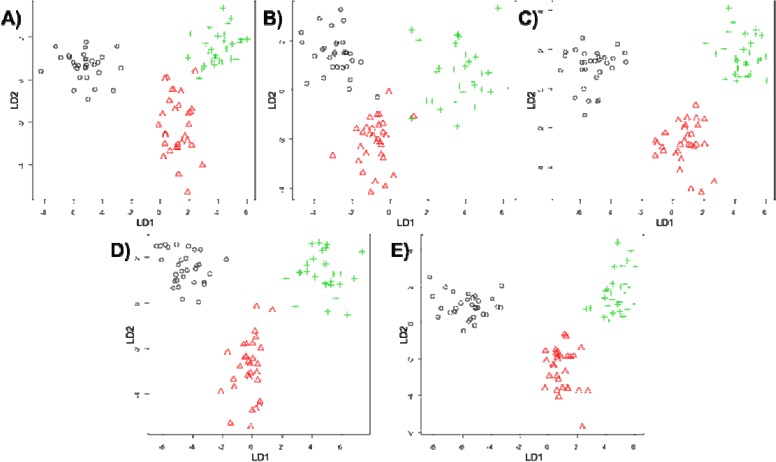
Principal components-linear discriminant analysis (PC-LDA) scores plots showing the comparison of the five different missing value substitutes—(**A**) zero; (**B**) mean; (**C**) median; (**D**) kNN and (**E**) RF—for the classification of normoxia (black circles), hypoxia (red triangles) and anoxia (green pluses).

An additional output from PC-LDA is the scores plot which shows the best group separation, and Figure 5 shows LD1 *vs.* LD2 scores. It is clear that the 3 different groups of samples are generally well separated; however it is also clear that median and RF imputation have resulted in the best separation, followed by zero and kNN replacement and lastly mean substitutions for missing values. A very similar pattern can be observed for the PLS-DA where median seems to be the best substitute for missing value (data not shown).

## 4. Conclusions

A summary of the class prediction results are shown in Table 1, and it is clear from this that RF performed the best. The RF imputation technique is followed by kNN, median and zero replacement methods that perform relatively well. By contrast, the mean imputation approach does not perform well which is an interesting observation, as this is the default method used by many researchers. An obvious question arises as to why this has been the case?

**Table 1:** Comparison of different missing values substitutions used for discriminant analysis.

			*PC-LDA*					*PLS-DA*		
	Zero	Mean	Median	kNN	RF	Zero	Mean	Median	kNN	RF
Classification rate (%) *	92.02	77.91	92.57	95.63	98.02	90.97	77.98	91.76	96.06	97.73
Number of latent variables (components) used	7	21	22	19	22	7	6	8	9	10

* based on classifying normoxia, hypoxia and anoxia.

The answer to the question is likely to be based on the concentration distribution profile of the metabolites collected. If a particular metabolite’s concentration follows a normal distribution then the mean imputation would be appropriate as the normal distribution has a Gaussian shape. Clearly in our case this is not the case as can be observed in Table 2. Almost half of the samples do not follow normal or near normal distribution as their values are highly positively or negatively skewed. Where for Normoxia samples the highest value (3.01) of positive skewness is recorded for *galactose/glucose*, followed by *glycerol* for Hypoxia with the highest value of 4.03 positive skewness, and finally *benzoic acid* with 4.15 positive skewness for Anoxia. Additional statistics such as percentage of missing values, mean, median, standard deviation, kurtosis and standard error can be found in Tables S2–S4.

**Table 2:** Skewness of 3 aeration environments (Normoxia, Hypoxia, Anoxia) for 52 metabolites.

Metabolite name	Normoxia	Hypoxia	Anoxia
*Glycine*	–2.59	0.98	0.70
*Lactate*	–0.25	0.13	–0.35
*Pyruvate*	0.14	0.25	1.54
*Valine*	0.95	0.63	0.97
*Leucine*	0.27	0.67	0.92
*Glycerol*	0.59	4.03	1.45
*Isoleucine*	0.12	1.29	1.05
*Leucine*	1.15	2.07	1.80
*Malonate*	–2.29	–0.61	–1.05
*Glycine*	–0.55	0.03	–0.09
*Phosphate*	1.29	0.78	1.01
*Threonine*	–0.66	0.76	1.19
*Alanine*	0.84	0.25	0.96
*Threonine*	0.35	1.24	1.05
*Succinate*	0.38	0.84	0.05
*Benzoic acid*	–0.60	–1.06	4.15
*Threitol/erythritol*	1.07	1.08	1.58
*Malate*	–0.09	0.91	0.45
*4-hydroxyproline*	–0.54	2.65	0.98
*Aspartate*	0.82	0.57	1.22
*4-aminobutyric acid*	0.70	0.91	0.03
*Aspartate*	–0.33	1.99	–0.32
*4-hydroxyproline*	0.10	0.00	0.86
*Xylitol*	0.99	1.00	0.68
*2-hydroxyglutaric acid*	0.89	1.24	0.22
*4-hydroxybenzoic acid*	–0.78	1.26	1.71
*Methionine*	0.18	1.23	1.10
*Creatinine*	0.32	0.49	–0.55
*Putrescine*	0.10	0.22	0.27
*Hypotaurine*	–0.07	0.27	–0.62
*Glutamate*	0.34	0.42	1.25
*2-oxoglutarate*	0.32	0.36	0.56
*Fructose*	–0.20	0.46	2.00
*Sorbose/fructose*	1.41	1.08	1.31
*Sorbitol/galactose /glucose*	1.20	1.08	0.93
*Sorbose/fructose*	1.55	1.56	1.34
*Glycerol 3-phosphate*	–0.68	0.80	–0.20
*Galactose/glucose*	2.28	1.33	1.99
*Galactose/glucose*	2.36	0.35	2.21
*Galactose/glucose*	3.01	1.52	1.97
*Citrate*	0.50	1.17	0.46
*N-acetyl aspartate*	–0.90	0.59	0.78
*Glucose*	2.15	0.63	2.39
*Scyllo-inositol*	0.93	0.55	1.53
*Lysine*	0.46	0.75	0.94
*Myo-inositol*	–1.65	0.29	–0.09
*Pantothenic acid*	1.58	0.38	0.72
*Tyramine/tyrosine*	0.82	0.85	1.04
*Hexadecanoic acid*	–0.86	1.57	1.57
*Octadecanoic acid*	–2.67	0.97	2.67
*Myo-inositol phosphate*	0.81	0.84	2.78
*Lactose/maltose*	0.33	1.21	0.83

Different conclusions were given when mean and median imputations were compared recently for data generated from direct infusion Fourier transform ion cyclotron resonance mass spectrometry (DI-FT-ICR-MS) [8], where mean and median showed nearly same results. This suggests that these DI-FT-ICR-MS data may had been normally distributed with little or no skews or outliers in the metabolite concentrations [6]. However, in our study we have shown that metabolites that display skewness can have a significant impact on the substitute of missing values and therefore, final output of the analysis. Where, skewness is an important feature that can articulate if the data are normally or near normally distributed where normal distribution has skewness of 0 or close to 0. As shown in this study not all metabolomics data follows normal distribution. Hence, we believe that careful analysis of distribution of the data prior to any analysis should be performed. Moreover, the authors recommended kNN, mean and median as imputation methods for missing values in metabolomics field. However, in our study we have shown that much better results can be accomplished when RF is used as a replacement method of missing values.

We have demonstrated that the selection method chosen to replace missing values may have a dramatic impact on GC-MS based metabolomics data, both for unsupervised and supervised learning. Therefore, the handling of missing values is an absolutely vital step in data pre-processing, as has been suggested previously in the literature [7], to which special consideration should be given. Moreover, we have shown that selecting the median metabolite level as a substitute of missing values is a substantially better option than the selection of the mean metabolite response, which is sensitive to metabolite distribution when data are skewed, as shown in this study. As observed in [8] when metabolite levels are normally distributed then both mean and median imputations will provide very similar results, but one cannot be guaranteed that this is always be the case. The median is less vulnerable to outliers than the arithmetic mean and is therefore considered to be more robust [6]. We conclude that before doing a missing value imputation that the distribution of the data be examined; conducting a skewness calculation or more simply observing the ratio of the mean and median of the distribution could identify this. We therefore believe that an awareness of these factors needs to be concomitant with an understanding of the data themselves, prior to any data analysis when missing values are observed [13], else the conclusions of the study could be inaccurate. In summary, in the case of GC-MS metabolomics data studied here our findings recommend that RF should be favored as an imputation of missing value over the other tested methods, as this method provides more robust and trustful results.

## Supplementary Files

Supplementary File 1 Supplementary Information (PDF, 982 KB)

## References

[1] Fiehn O. (2001). Combining genomics, metabolome analysis, and biochemical modelling to understand metabolic networks. Comp. Funct. Genom..

[2] Goodacre R., Vaidyanathan S., Dunn W.B., Harrigan G.G., Kell D.B. (2004). Metabolomics by numbers: Acquiring and understanding global metabolite data. Trends Biotechnol..

[3] Jenkins S., Fischer S.M., Chen L., Sana T.R. (2013). Global LC/MS metabolomics profiling of calcium stressed and immunosuppressant drug treated saccharomyces cerevisiae. Metabolites.

[4] Kassama Y., Xu Y., Dunn W.B., Geukens N., Anne J., Goodacre R. (2010). Assessment of adaptive focused acoustics *versus* manual vortex/freeze-thaw for intracellular metabolite extraction from Streptomyces lividans producing recombinant proteins using GC-MS and multi-block principal component analysis. Analyst.

[5] Begley P., Francis-McIntyre S., Dunn W.B., Broadhurst D.I., Halsall A., Tseng A., Knowles J., Goodacre R., Kell D.B. (2009). Development and performance of a gas chromatography-time-of-flight mass spectrometry analysis for large-scale nontargeted metabolomic studies of human serum. Anal. Chem..

[6] Steuer R., Morgenthal K., Weckwerth W., Selbig J. (2007). A gentle guide to the analysis of metabolomic data. Methods Mol. Biol..

[7] Goodacre R., Broadhurst D., Smilde A.K., Kristal B.S., Baker J.D., Beger R., Bessant C., Connor S., Calmani G., Craig A. (2007). Proposed minimum reporting standards for data analysis in metabolomics. Metabolomics.

[8] Hrydziuszko O., Viant M.R. (2011). Missing values in mass spectrometry based metabolomics: An undervalued step in the data processing pipeline. Metabolomics.

[9] Xia J., Psychogios N., Young N., Wishart D.S. (2009). MetaboAnalyst: A web server for metabolomic data analysis and interpretation. Nucleic Acids Res..

[10] Schafer J.L., Graham J.W. (2002). Missing Data: Our View of the State of the Art. Psychol. Methods.

[11] De Ligny C.L., Nieuwdorp G.H.E., Brederode W.K., Hammers W.E., Vanhouwelingen J.C. (1981). An Application of factor analysis with missing data. Technometrics.

[12] Duran A.L., Yang J., Wang L.J., Sumner L.W. (2003). Metabolomics spectral formatting, alignment and conversion tools (MSFACTs). Bioinformatics.

[13] Little R.J.A., Rubin D.B. (1987). Statistical Analysis with Missing Data.

[14] Shrive F.M., Stuart H., Quan H., Ghali W.A. (2006). Dealing with missing data in a multi-question depression scale: A comparison of imputation methods. BMC Med. Res. Methodol..

[15] Stacklies W., Redestig H., Scholz M., Walther D., Selbig J. (2007). pcaMethods—A bioconductor package providing PCA methods for incomplete data. Bioinformatics.

[16] Walczak B., Massart D.L. (2001). Dealing with missing data: Part I. Chemom. Intell. Lab..

[17] Walczak B., Massart D.L. (2001). Dealing with missing data: Part II. Chemom. Intell. Lab..

[18] Steinfath M., Groth D., Lisec J., Selbig J. (2008). Metabolite profile analysis: From raw data to regression and classification. Physiol. Plant..

[19] Steuer R. (2006). On the analysis and interpretation of correlations in metabolomic data. Brief. Bioinform..

[20] Hair J.F., Black W.C., Babin B.J., Anderson R.E. (2010). Multivariate Data Analysis.

[21] Kotze H.L., Armitage E.G., Sharkey K.J., Allwood J.W., Dunn W.B., Williams K.J., Goodacre R. (2013). A novel untargeted metabolomics correlation-based network analysis incorporating human metabolic reconstructions. BMC Syst. Biol..

[22] Troyanskaya O., Cantor M., Sherlock G., Brown P., Hastie T., Tibshirani R., Botstein D., Altman R.B. (2001). Missing value estimation methods for DNA microarrays. Bioinformatics.

[23] Stekhoven D.J., Buehlmann P. (2012). MissForest-non-parametric missing value imputation for mixed-type data. Bioinformatics.

[24] Teng Q., Huang W., Collette T.W., Ekman D.R., Tan C. (2009). A direct cell quenching method for cell-culture based metabolomics. Metabolomics.

[25] Wedge D.C., Allwood J.W., Dunn W., Vaughan A.A., Simpson K., Brown M., Priest L., Blackhall F.H., Whetton A.D., Dive C. (2011). Is serum or plasma more appropriate for intersubject comparisons in metabolomic studies? An assessment in patients with small-cell lung cancer. Anal. Chem..

[26] Dunn W.B., Broadhurst D., Begley P., Zelena E., Francis-McIntyre S., Anderson N., Brown M., Knowles J.D., Halsall A., Haselden J.N. (2011). Procedures for large-scale metabolic profiling of serum and plasma using gas chromatography and liquid chromatography coupled to mass spectrometry. Nat. Protoc..

[27] Pope G.A., MacKenzie D.A., Defemez M., Aroso M.A.M.M., Fuller L.J., Mellon F.A., Dunn W.B., Brown M., Goodacre R., Kell D.B. (2007). Metabolic footprinting as a tool for discriminating between brewing yeasts. Yeast.

[28] Kopka J., Schauer N., Krueger S., Birkemeyer C., Usadel B., Bergmuller E., Dormann P., Weckwerth W., Gibon Y., Stitt M. (2005). GMD@CSB.DB: The golm metabolome database. Bioinformatics.

[29] Sumner L.W., Amberg A., Barrett D., Beale M.H., Beger R., Daykin C.A., Fan T.W.M., Fiehn O., Goodacre R., Griffin J.L. (2007). Proposed minimum reporting standards for chemical analysis. Metabolomics.

[30] Team R.D.C. (2008). R: A Language and Environment for Statistical Computing.

[31] Varmuza K., Filzmoser P. (2009). Introduction to Multivariate Statistical Analysis in Chemometrics.

[32] Venables W.N., Ripley B.D. (2002). Modern Applied Statistics with S.

[33] Adler D., Murdoch D. rgl: 3D Visualization Device System (OpenGL).

[34] Dejean S., Gonzalez I., Cao K.-A.L., Monget P., Coquery J., Yao F., Liquet B., Rohart F. mixOmics: Omics Data Integration Project.

[35] Hastie T., Tibshirani R., Narasimhan B., Chu G. Impute: Imputation for Microarray Data, 1.39.0. http://bioconductor.org/packages/devel/bioc/manuals/impute/man/impute.pdf2014.

[36] Gentleman R.C., Carey V.J., Bates D.M., Bolstad B., Dettling M., Dudoit S., Ellis B., Gautier L., Ge Y.C., Gentry J. (2004). Bioconductor: Open software development for computational biology and bioinformatics. Genome Biol..

[37] Stekhoven D.J. missForest: Nonparametric Missing Value Imputation using Random Forest, 1.4. http://cran.r-project.org/web/packages/mixOmics/index.html.

[38] Brereton R.G. (2006). Consequences of sample size, variable selection, and model validation and optimisation, for predicting classification ability from analytical data. TrAC.

[39] Van den Berg R.A., Hoefsloot H.C.J., Westerhuis J.A., Smilde A.K., van der Werf M.J. (2006). Centering, scaling, and transformations: Improving the biological information content of metabolomics data. BMC Genomics.

[40] Bro R., Smilde A.K. (2003). Centering and scaling in component analysis. J. Chemom..

[41] Breiman L. (2001). Random forests. Mach. Learn..

[42] Duda R.O., Hart P.E., Stork D.G. (2001). Unsupervised Learning and Clustering. Pattern Classification.

[43] Pearson K. (1901). On lines and planes of closest fit to systems of points in space. Philos. Mag..

[44] Hotelling H. (1933). Analysis of a complex of statistical variables into principal components. J. Educ. Psychol..

[45] Jolliffe I.T. (2002). Principal Component Analysis..

[46] Burman P. (1989). A comparative study of ordinary cross-validation, v-fold cross-validation and the repeated learning-testing methods. Biometrika.

[47] Kohavi R. (1995). A study of cross-validation and bootstrap for accuracy estimation and model selection. Proceedings of the 14th International Joint Conference on Artificial Intelligence.

[48] Jain A.K., Duin R.P.W., Mao J.C. (2000). Statistical pattern recognition: A review. IEEE Trans. Pattern Anal. Mach. Intell..

[49] Jain A.K., Murty M.N., Flynn P.J. (1999). Data clustering: A review. ACM Comput. Surv..

[50] Everitt B. (1974). Cluster Analysis.

[51] Szekely G.J., Rizzo M.L. (2005). Hierarchical clustering via joint between-within distances: Extending Ward’s minimum variance method. J. Classif..

[52] Ward J.H. (1963). Hierarchical grouping to optimize an objective function. JASA.

[53] Hastie T., Tibshirani R., Friedman J. (2009). The Elements of Statistical Learning Data Mining, Inference, and Prediction.

[54] Manly B.F.J. (1986). Multivariate Statistical Methods: A Primer.

[55] Dixon W.J. (1975). Biomedical Computer Programs.

[56] Goodacre R., Timmins E.M., Burton R., Kaderbhai N., Woodward A.M., Kell D.B., Rooney P.J. (1998). Rapid identification of urinary tract infection bacteria using hyperspectral whole-organism fingerprinting and artificial neural networks. Microbiology.

[57] Macfie H.J.H., Gutteridge C.S., Norris J.R. (1978). Use of canonical variates analysis in differentiation of bacteria by pyrolysis gas-liquid chromatography. Microbiology.

[58] Barker M., Rayens W. (2003). Partial least squares for discrimination. J. Chemom..

[59] Gromski P.S., Xu Y., Correa E., Ellis D.I., Turner M.L., Goodacre R. (2014). A comparative investigation of modern feature selection and classification approaches for the analysis of mass spectrometry data. Anal. Chim. Acta.

[60] Haenlein M., Kaplan A.M. (2004). A beginner’s guide to partial least squares analysis. Und. Stat..

[61] Wold S., Sjostrom M., Eriksson L. (2001). PLS-regression: A basic tool of chemometrics. Chemom. Intell. Lab. Syst..

[62] Efron B. (1979). 1977 rietz lecture. bootstrap methods: Another look at the Jackknife. Ann. Stat..

[63] Efron B., Gong G. (1983). A leisurely look at the bootstrap, the jackknife, and cross-validation. Am. Stat..

[64] Kotze H.L. (2012). System Biology of Chemotherapy in Hypoxia Environments.

[65] Xu Y., Goodacre R. (2012). Multiblock principal component analysis: An efficient tool for analyzing metabolomics data which contain two influential factors. Metabolomics.

[66] Brereton R.G., Lloyd G.R. (2014). Partial least squares discriminant analysis: Taking the magic away. J. Chemom..

