# Supplementary material for: Influence of Missing Values Substitutes on Multivariate Analysis of Metabolomics Data
Source: Metabolites. 2014 Jun 16;4(2):433–52. doi: 10.3390/metabo4020433 (PMC4101515; doi:10.3390/metabo4020433)
Supplement: Supplementary File 1 — Supplementary Information (PDF, 982 KB) [file metabolites-04-00433-s001.pdf]

Supplementary Information

Influence of Missing Values Substitutes on Multivariate Analysis of Metabolomics Data

1. Principal Components Analysis (PCA)

As discussed in the main text after PCA, the original data are reduced to a number of significant PCs. Figure S1A displays the PCA procedure where the original data are converted into significant PCs by a decomposition of the data matrix ***X*** into two matrices, where the series of columns are represented as scores, ***T***, and the series of rows are termed loadings, ***P***. Additionally, both are orthogonal. The loadings can be used to understand which input variables are important as the weight for each original variable when computing the PC. Moreover, the first vectors demonstrating both series (scores and loadings) are called then eigenvectors of the first PC, the second vectors are named the eigenvectors of second PC and so on [1,2].

Figure S1. (**A**) Matrix structure for PCA, where loadings summarise variation in variables and scores summarise variation in samples. This results in an approximated matrix with reduced noise ($\boldsymbol{T} = \boldsymbol{P} \times \boldsymbol{X}$) [1]; (**B**) A graphical illustration of multiblock PCA.

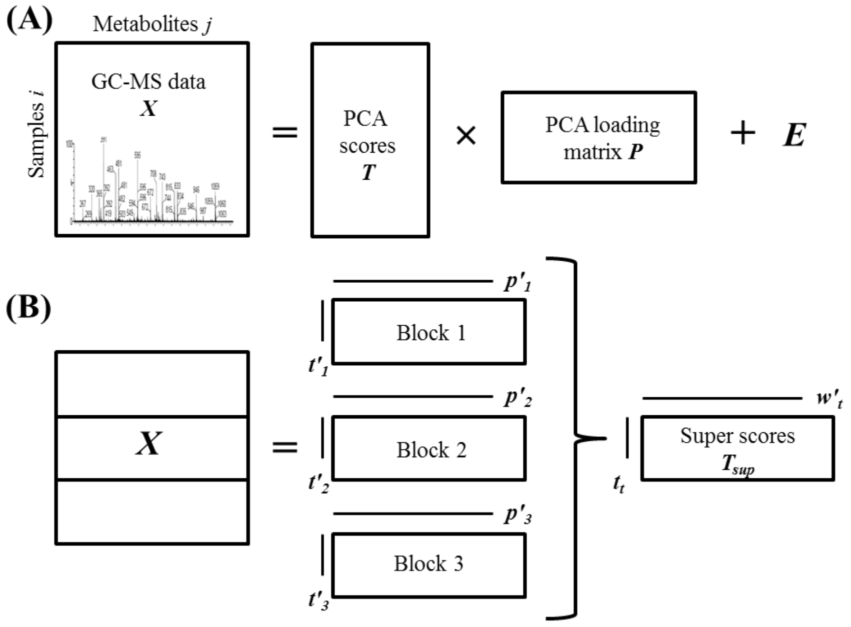

2. Multiblock PCA

Figure S1A displays the scheme for the PCA method, which is presented here for comparing with the multiblock extension of the method. Where, a data matrix with descriptor ***X*** can be considered here as a typical output of GC-MS with variables representing different metabolites. In contrast to PCA as shown in Figure S1B multiblock PCA associates three blocks of descriptors, the variables (metabolites) measured for samples that correspond to three cases normoxia, hypoxia and anoxia as described in manuscript, where the consensus direction among all the of them is pursued. As the method is closely relate to PCA, hence, latent variable and scores are calculated for each of the blocks, in the next step of calculation these values are combined into one super score block. The final output displays the consensus of all projections comprised in super scores T_{sup}, where weight w'_t highlight the significance

of each projection. This method can be used when typical PCA fails and has been previously used in the analysis of metabolomics data which contain two influential factors [3–5].

3. Hierarchical Cluster Analysis (HCA)

Hierarchical clustering belongs to the group of unsupervised technique, where objects are ordered into a hierarchical structure. The method that we employ is agglomerative HCA and is based on the stepwise procedure requiring an amalgamation of the observations into a cluster. The clusters are displayed as a dendrogram which is a graphical representation of a treelike graph structure, where the vertical line shows distances between individuals in hierarchy and horizontal lines depict the grouping of observations according to their characteristics; *i.e.*, nearest neighbours. The creation of the dendrogram starts with all individuals separate as a single group, thereafter the closest observations are merged together into a single cluster and so on until all objects are merged into the one final group. The process of generation of these clusters is achieved by the measure of distances of each individual towards other individuals. Hence, form a hierarchy of clusters from small to the final merged cluster. Generated cluster provides useful information about the data which are interpreted by the analyst [6,7].

4. Statistics on Missing Values Substitutes

Figure S2. Typical graphical representation of high dimensional data. Where the unsupervised approaches (PCA, HCA) use only X input data for the analysis and supervised approaches (PC-LDA, PLS-DA) which use both data sets for the analysis by associating Y (dependent variable) output data that represents the original grouping information *i.e.* classes (groups, traits) with each of the inputs X.

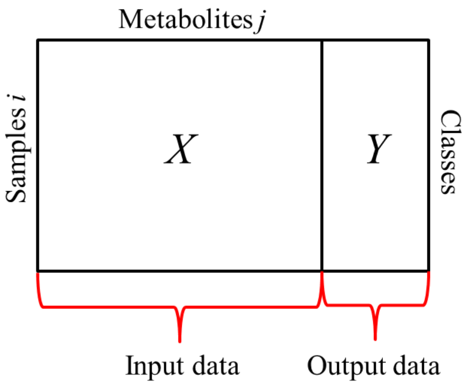

In this study multiblock PCA under the name CPCA was implemented as described [4]. Moreover, this analysis were performed using MATLAB® version 2010a (MathWorks Inc., Natick, Massachusetts, USA).

Figure S3. Evaluation of five different missing value substitutes: (**A**) zero; (**B**) mean; (**C**) median, (**D**) kNN and (**E**) RF imputations, established on their outcomes on PCA scores plots for the 9 classes analysed with GC-MS.

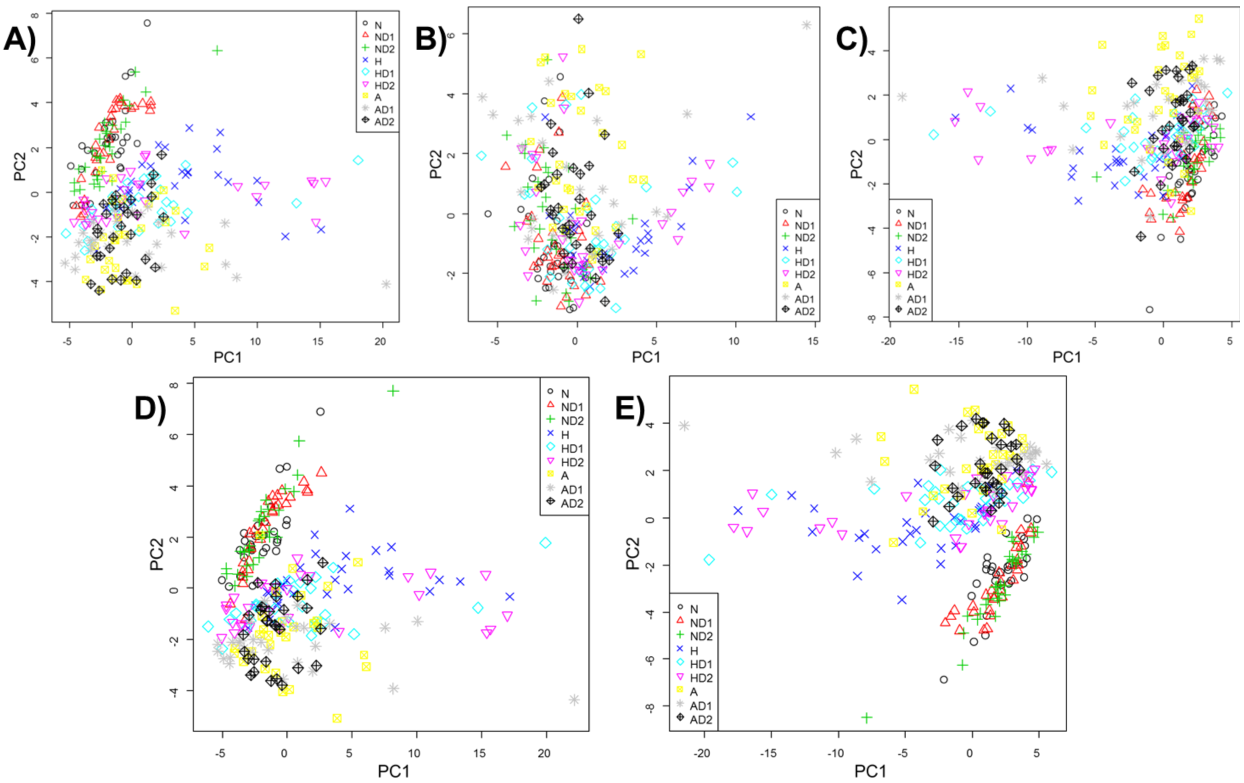

Figure S4. The block scores plot of the CPCA-W performed on the 3 × 3 experiment design. Symbols characterized as: normoxia (black circles), hypoxia (red triangles) and anoxia (green pluses).

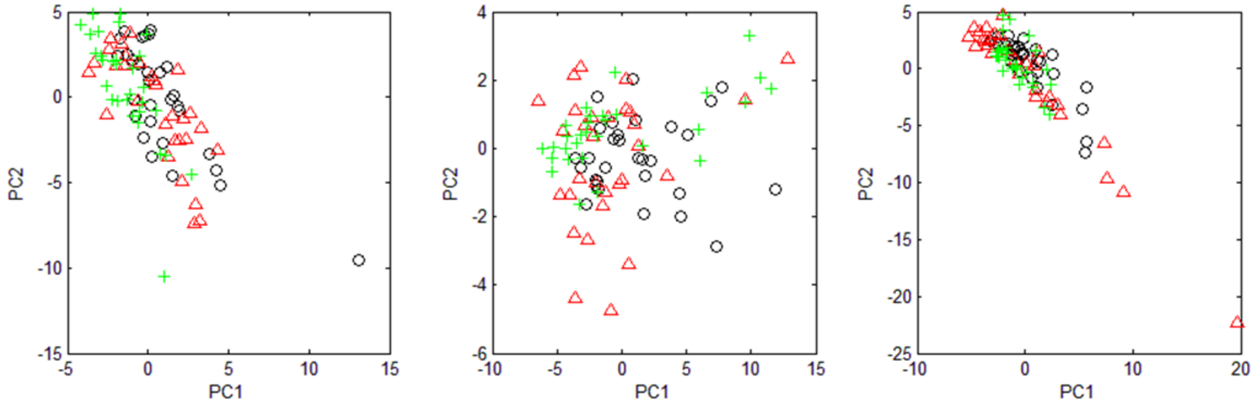

Figure S5. Three-dimensional visualization of PCA scores plots. Evaluation of five different missing value substitutes: (**A**) zero; (**B**) mean; (**C**) median; (**D**) kNN and (**E**) RF established on their outcome on PCA scores plots. Symbols characterized as: normoxia (black circles), hypoxia (red triangles) and anoxia (green pluses).

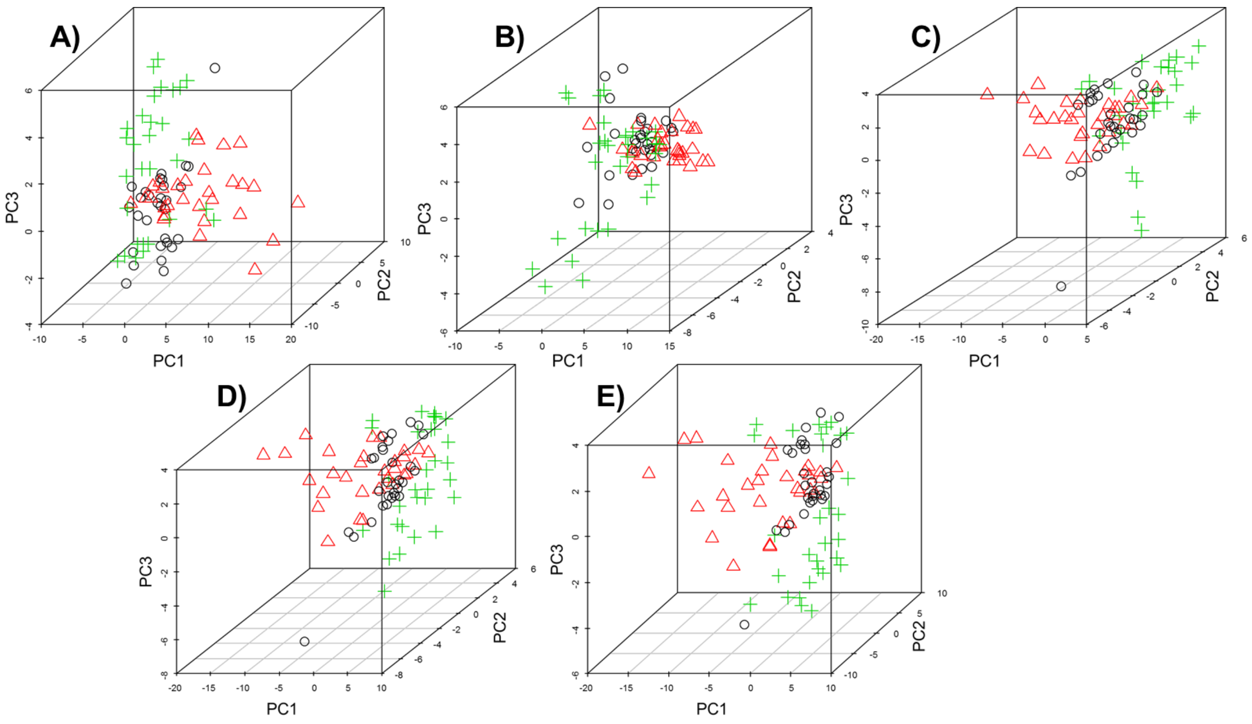

Table S1. Purity (%) for HCA based on Euclidean distance with "Wards" linkage for five different value substitutes: zero, mean, (C) median, kNN and RF.

	Hypoxia	*Normoxia*	*Anoxia*
**Zero**	86.37	74.36	93.10
**Mean**	87.50	48.84	64.52
**Median**	85.00	90.63	65.79
**kNN**	85.71	96.42	58.33
**RF**	79.17	100	64.10

Table S2. Summary statistics of 52 metabolite peaks for Normoxia.

Metabolite Name	MV(%) *	Mean	Sd *	Median	Skew	Kurtosis	SE *
Glycine	0	0.86	0.16	0.91	−2.59	5.53	0.03
Lactate	0	1.67	0.31	1.72	−0.25	−1.19	0.06
Pyruvate	0	0.03	0.01	0.03	0.14	−0.75	2.E-03
Valine	73.33	1.E-03	9.E-04	1.E-03	0.95	−0.53	3.E-04
Leucine	16.66	0.07	0.02	0.07	0.27	−0.83	4.E-03
Glycerol	0	0.05	0.02	0.04	0.59	−1.09	3.E-03
Isoleucine	10	0.10	0.03	0.09	0.12	−1.23	5.E-03
Leucine	23.33	−0.39	0.01	6.E-03	1.15	0.68	1.E-03
Malonate	3.33	−0.58	0.05	0.51	−2.29	5.94	9.E-03
Glycine	20	−0.78	0.01	0.12	−0.55	0.65	2.E-03

Table S2. *Cont.*

Metabolite Name	MV(%) *	Mean	Sd *	Median	Skew	Kurtosis	SE *
Phosphate	6.66	−0.98	1.28	4.18	1.29	0.75	0.24
Threonine	13.33	−1.17	0.01	0.03	−0.66	−0.61	2.E-03
Alanine	0	−1.37	0.02	0.04	0.84	−0.11	4.E-03
Threonine	60	2.E-03	1.E-03	2.E-03	0.35	−1.67	4.E-04
Succinate	30	0.01	2.E-03	0.01	0.38	−0.99	5.E-04
Benzoic acid	3.33	0.01	2.E-03	0.01	−0.60	0.33	4.E-04
Threitol/erythritol	10	0.03	8.E-03	0.03	1.07	1.54	2.E-03
Malate	0	0.02	6.E-03	0.02	−0.09	−1.13	1.E-03
4-hydroxyproline	0	0.05	9.E-03	0.05	−0.54	−0.49	2.E-03
Aspartate	13.33	0.02	5.E-03	0.02	0.82	1.47	1.E-03
4-aminobutyric acid	33.33	8.E-03	2.E-03	7.E-03	0.70	0.63	4.E-04
Aspartate	90	4.E-03	4.E-04	4.E-03	−0.33	−2.33	2.E-04
4-hydroxyproline	20	0.07	0.02	0.07	0.10	−0.73	4.E-03
Xylitol	6.66	7.E-03	2.E-03	8.E-03	0.99	1.30	3.E-04
2-hydroxyglutaric acid	20	5.E-03	1.E-03	5.E-03	0.89	1.47	3.E-04
4-hydroxybenzoic acid	0	0.02	3.E-03	0.02	−0.78	2.30	6.E-04
Methionine	30	7.E-03	3.E-03	7.E-03	0.18	−1.37	6.E-04
Creatinine	70	4.E-03	1.E-03	3.E-03	0.32	−1.62	5.E-04
Putrescine	10	0.12	0.04	0.13	0.10	−0.45	7.E-03
Hypotaurine	0	0.02	5.E-03	0.02	−0.07	−0.06	8.E-04
Glutamate	0	0.54	0.23	0.51	0.34	0.56	0.04
2-oxoglutarate	6.66	0.00	1.E-03	3.E-03	0.32	−0.63	2.E-04
Fructose	6.66	7.E-03	2.E-03	7.E-03	−0.20	1.12	4.E-04
Sorbose/fructose	0	0.16	0.07	0.14	1.41	2.84	0.01
Sorbitol/galactose /glucose	3.33	0.03	0.01	0.02	1.20	1.97	2.E-03
Sorbose/fructose	0	0.06	0.03	0.05	1.55	3.47	5.E-03
Glycerol 3-phosphate	3.33	0.02	4.E-03	0.02	−0.68	1.02	8.E-04
Galactose/glucose	0	0.06	0.06	0.05	2.28	6.67	0.01
Galactose/glucose	0	0.02	0.02	0.01	2.36	7.12	3.E-03
Galactose/glucose	3.33	0.01	0.02	0.01	3.01	10.77	3.E-03
Citrate	0	0.07	0.02	0.06	0.50	−0.59	3.E-03
N-acetyl aspartate	16.66	0.01	9.E-04	6.E-03	−0.90	1.65	2.E-04
Glucose	3.33	0.02	0.02	0.02	2.15	5.96	4.E-03
Scyllo-inositol	33.33	6.E-03	1.E-03	6.E-03	0.93	1.27	3.E-04
Lysine	83.33	5.E-03	3.E-03	5.E-03	0.46	−1.58	1.E-03
Myo-inositol	3.33	0.65	0.12	0.67	−1.65	4.73	0.02
Pantothenic acid	13.33	1.E-03	5.E-04	1.E-03	1.58	2.96	1.E-04
Tyramine/tyrosine	23.33	7.E-03	4.E-03	6.E-03	0.82	−0.13	9.E-04
Hexadecanoic acid	0.00	0.02	5.E-03	0.02	−0.86	3.85	9.E-04
Octadecanoic acid	3.33	0.11	0.02	0.11	−2.67	9.84	4.E-03
Myo-inositol phosphate	40	1.E-03	5.E-04	1.E-03	0.81	0.70	1.E-04
Lactose/maltose	16.66	2.E-03	9.E-04	2.E-03	0.33	−1.48	2.E-04

* MV-percentage of missing values, sd-standard deviation, SE-standard error.

Table S3. Summary of statistics 52 metabolite peaks for Hypoxia.

Metabolite Name	MV(%) *	Mean	Sd *	Median	Skew	Kurtosis	SE *
Glycine	3.33	0.94	0.04	0.93	0.98	2.26	7.E-03
Lactate	3.33	2.14	0.60	2.06	0.13	0.38	0.11
Pyruvate	3.33	0.03	0.01	0.03	0.25	–0.76	2.E-03
Valine	33.33	6.E-03	3.E-03	5.E-03	0.63	–1.03	7.E-04
Leucine	10	0.17	0.07	0.17	0.67	0.01	0.01
Glycerol	0	0.12	0.13	0.08	4.03	17.1	0.02
Isoleucine	0	0.18	0.08	0.17	1.29	2.25	0.01
Leucine	6.67	0.05	0.06	0.02	2.07	3.65	0.01
Malonate	3.33	0.52	0.03	0.52	–0.61	2.79	6.E-03
Glycine	16.67	0.23	0.04	0.24	0.03	–0.32	8.E-03
Phosphate	6.67	4.45	1.97	4.55	0.78	0.43	0.37
Threonine	3.33	0.06	0.03	0.05	0.76	–0.49	5.E-03
Alanine	23.33	0.04	0.02	0.04	0.25	–0.66	3.E-03
Threonine	40	0.01	0.01	0.01	1.24	0.33	3.E-03
Succinate	33.33	0.02	6.E-03	0.02	0.84	–0.38	1.E-03
Benzoic acid	0	0.01	3.E-03	0.01	–1.06	2.05	6.E-04
Threitol/erythritol	0	0.06	0.03	0.05	1.08	0.53	5.E-03
Malate	0	0.02	5.E-03	0.02	0.91	0.80	8.E-04
4-hydroxyproline	0	0.06	0.02	0.05	2.65	8.18	4.E-03
Aspartate	3.33	0.03	0.02	0.03	0.57	–0.33	3.E-03
4-aminobutyric acid	13.33	0.01	7.E-03	8.E-03	0.91	0.21	1.E-03
Aspartate	53.33	0.03	0.03	0.01	1.99	3.04	8.E-03
4-hydroxyproline	6.67	0.09	0.03	0.09	0.00	–1.50	6.E-03
Xylitol	6.67	0.02	5.E-03	0.01	1.00	0.40	9.E-04
2-hydroxyglutaric acid	16.67	8.E-03	3.E-03	7.E-03	1.24	0.83	5.E-04
4-hydroxybenzoic acid	0	0.03	4.E-03	0.03	1.26	1.25	7.E-04
Methionine	26.67	0.02	0.01	0.01	1.23	1.13	2.E-03
Creatinine	53.33	0.01	3.E-03	6.E-03	0.49	–0.89	9.E-04
Putrescine	30	0.12	0.04	0.12	0.22	–0.97	9.E-03
Hypotaurine	0	0.02	5.E-03	0.02	0.27	–0.70	9.E-04
Glutamate	0	1.19	0.59	1.08	0.42	–0.90	0.11
2-oxoglutarate	23.33	1.E-03	4.E-04	1.E-03	0.36	–1.02	8.E-05
Fructose	16.67	5.E-03	2.E-03	5.E-03	0.46	–1.06	4.E-04
Sorbose/fructose	3.33	0.40	0.22	0.34	1.08	0.52	0.04
Sorbitol/galactose /glucose	3.33	0.06	0.03	0.06	1.08	0.67	6.E-03
Sorbose/fructose	0	0.16	0.10	0.13	1.56	2.47	0.02
Glycerol 3-phosphate	0	0.05	0.02	0.05	0.80	0.05	3.E-03
Galactose/glucose	0	0.06	0.05	0.05	1.33	1.12	1.E-02
Galactose/glucose	3.33	0.01	8.E-03	0.01	0.35	–0.99	1.E-03
Galactose/glucose	0	0.01	0.01	9.E-03	1.52	1.83	2.E-03
Citrate	0	0.03	9.E-03	0.03	1.17	0.79	2.E-03
N-acetyl aspartate	16.67	4.E-03	1.E-03	4.E-03	0.59	–0.51	2.E-04
Glucose	0	0.01	9.E-03	0.01	0.63	–0.66	2.E-03
Scyllo-inositol	43.33	5.E-03	1.E-03	5.E-03	0.55	–1.21	3.E-04

Table S3. *Cont.*

Metabolite Name	MV(%) *	Mean	Sd *	Median	Skew	Kurtosis	SE *
Lysine	33.33	0.03	0.02	0.03	0.75	–0.05	5.E-03
Myo-inositol	3.33	0.74	0.12	0.73	0.29	–0.66	0.02
Pantothenic acid	0	1.E-03	5.E-04	1.E-03	0.38	–0.62	9.E-05
Tyramine/tyrosine	0	0.03	0.02	0.02	0.85	–0.36	3.E-03
Hexadecanoic acid	0	0.03	6.E-03	0.03	1.57	2.60	1.E-03
Octadecanoic acid	0	0.13	0.02	0.13	0.97	1.14	3.E-03
Myo-inositol phosphate	3.33	5.E-03	4.E-03	3.E-03	0.84	–0.75	8.E-04
Lactose/maltose	6.67	4.E-03	3.E-03	2.E-03	1.21	0.45	6.E-04

* MV-percentage of missing values, sd-standard deviation, SE-standard error.

Table S4. Summary statistics of 52 metabolite peaks for Anoxia.

Metabolite Name	MV(%) *	Mean	sd *	Median	Skew	Kurtosis	SE *
Glycine	0	0.94	0.04	0.93	0.70	–0.30	7.E-03
Lactate	0	1.82	0.55	1.83	–0.35	0.20	0.10
Pyruvate	23.33	0.01	8.E-03	9.E-03	1.54	1.79	2.E-03
Valine	40	4.E-03	2.E-03	4.E-03	0.97	–0.26	5.E-04
Leucine	16.67	0.12	0.05	0.11	0.92	–0.40	1.E-02
Glycerol	0	0.07	0.03	0.07	1.45	1.78	6.E-03
Isoleucine	10	0.12	0.05	0.11	1.05	0.35	1.E-02
Leucine	23.33	0.02	0.02	0.01	1.80	1.82	4.E-03
Malonate	3.33	0.47	0.05	0.48	–1.05	0.51	9.E-03
Glycine	20	0.14	0.04	0.13	–0.09	0.02	9.E-03
Phosphate	3.33	4.86	2.24	3.66	1.01	–0.03	0.42
Threonine	3.33	0.05	0.02	0.04	1.19	0.57	4.E-03
Alanine	53.33	0.02	0.01	0.02	0.96	0.03	3.E-03
Threonine	80	0.02	0.02	0.01	1.05	–0.55	8.E-03
Succinate	46.67	0.02	6.E-03	0.02	0.05	–1.60	2.E-03
Benzoic acid	0	0.02	0.02	0.01	4.15	17.20	4.E-03
Threitol/erythritol	0	0.04	0.02	0.03	1.58	2.33	3.E-03
Malate	0	0.01	6.E-03	9.E-03	0.45	–0.91	1.E-03
4-hydroxyproline	0	0.07	0.02	0.06	0.98	0.91	3.E-03
Aspartate	10	0.02	9.E-03	0.02	1.22	1.32	2.E-03
4-aminobutyric acid	63.33	7.E-03	2.E-03	6.E-03	0.03	–1.21	7.E-04
Aspartate	90	0.02	0.01	0.02	–0.32	–2.33	8.E-03
4-hydroxyproline	30	0.05	0.02	0.04	0.86	–0.29	5.E-03
Xylitol	0	0.01	4.E-03	0.01	0.68	0.54	7.E-04
2-hydroxyglutaric acid	16.67	0.02	1.E-02	0.02	0.22	–0.77	2.E-03
4-hydroxybenzoic acid	0	0.03	7.E-03	0.03	1.71	3.78	1.E-03
Methionine	40	0.01	9.E-03	0.01	1.10	–0.10	2.E-03
Creatinine	76.67	5.E-03	2.E-03	5.E-03	–0.55	–1.57	7.E-04
Putrescine	20	0.08	0.04	0.09	0.27	–0.41	8.E-03
Hypotaurine	30	0.03	0.01	0.03	–0.62	–0.40	2.E-03
Glutamate	0	0.61	0.47	0.42	1.25	0.21	9.E-02

Table S4. *Cont.*

Metabolite Name	MV(%) *	Mean	sd *	Median	Skew	Kurtosis	SE *
2-oxoglutarate	50	1.E-03	4.E-04	9.E-04	0.56	–0.95	1.E-04
Fructose	30	4.E-03	3.E-03	3.E-03	2.00	3.91	6.E-04
Sorbose/fructose	0	0.22	0.12	0.18	1.31	1.40	2.E-02
Sorbitol/galactose /glucose	0	0.04	0.02	0.03	0.93	0.26	3.E-03
Sorbose/fructose	10	0.08	0.05	0.07	1.34	1.78	9.E-03
Glycerol 3-phosphate	10	0.04	0.03	0.05	–0.20	–1.42	5.E-03
Galactose/glucose	0	0.04	0.06	0.02	1.99	2.76	0.01
Galactose/glucose	6.67	0.02	0.02	7.E-03	2.21	4.12	5.E-03
Galactose/glucose	6.67	1.E-02	0.01	4.E-03	1.97	2.63	3.E-03
Citrate	6.67	0.01	4.E-03	0.01	0.46	–0.58	7.E-04
N-acetyl aspartate	46.67	1.E-03	7.E-04	1.E-03	0.78	–0.47	2.E-04
Glucose	0	0.02	0.03	0.01	2.39	5.29	6.E-03
Scyllo-inositol	23.33	6.E-03	5.E-03	5.E-03	1.53	3.14	1.E-03
Lysine	53.33	0.02	0.01	0.01	0.94	–0.59	4.E-03
Myo-inositol	0	0.84	0.43	0.89	–0.09	–1.31	0.08
Pantothenic acid	13.33	1.E-03	4.E-04	1.E-03	0.72	1.08	7.E-05
Tyramine/tyrosine	6.67	0.01	0.01	0.01	1.04	–0.02	2.E-03
Hexadecanoic acid	0	0.03	6.E-03	0.03	1.57	3.73	1.E-03
Octadecanoic acid	3.33	0.15	0.04	0.14	2.67	7.96	7.E-03
Myo-inositol phosphate	50	2.E-03	2.E-03	2.E-03	2.78	6.84	6.E-04
Lactose/maltose	26.67	3.E-03	2.E-03	2.E-03	0.83	–0.79	4.E-04

* MV-percentage of missing values, sd-standard deviation, SE-standard error.

References

1. Jolliffe, I.T. *Principal Component Analysis*, 2nd ed.; Springer: New York, NY, USA, 2002; p. 487.
2. Martinez, A.M.; Kak, A.C. PCA *versus* LDA. *IEEE Trans. Pattern Anal. Mach. Intell.* **2001**, *23*, 228–233.
3. Smilde, A.K.; Westerhuis, J.A.; de Jong, S. A framework for sequential multiblock component methods. *J. Chemom.* **2003**, *17*, 323–337.
4. Westerhuis, J.A.; Kourti, T.; MacGregor, J.F. Analysis of multiblock and hierarchical PCA and PLS models. *J. Chemom.* **1998**, *12*, 301–321.
5. Xu, Y.; Goodacre, R. Multiblock principal component analysis: An efficient tool for analyzing metabolomics data which contain two influential factors. *Metabolomics* **2012**, *8*, S37–S51.
6. Everitt, B. *Cluster Analysis*; Heinemann Educational Books: London, UK, 1974; p. 122.
7. Jain, A.K.; Murty, M.N.; Flynn, P.J. Data clustering: A review. *ACM Comput. Surv.* **1999**, *31*, 264–323.

© 2014 by the authors; licensee MDPI, Basel, Switzerland. This article is an open access article distributed under the terms and conditions of the Creative Commons Attribution license (http://creativecommons.org/licenses/by/3.0/).
